# Corneal Nerve Regeneration after Self-Retained Cryopreserved Amniotic Membrane in Dry Eye Disease

**DOI:** 10.1155/2017/6404918

**Published:** 2017-08-15

**Authors:** Thomas John, Sean Tighe, Hosam Sheha, Pedram Hamrah, Zeina M. Salem, Anny M. S. Cheng, Ming X. Wang, Nathan D. Rock

**Affiliations:** ^1^Thomas John Vision Institute, Tinley Park, Cook County, IL, USA; ^2^Loyola University at Chicago, Maywood, Chicago, IL, USA; ^3^Ocular Surface Center and TissueTech, Inc., Miami, FL, USA; ^4^Florida International University Herbert Wertheim College of Medicine, Miami, FL, USA; ^5^Research Institute of Ophthalmology, Cairo, Egypt; ^6^Boston Image Reading Center, Tufts Medical Center, Tufts University School of Medicine, Boston, MA, USA; ^7^Center for Translational Ocular Immunology, Department of Ophthalmology, Tufts Medical Center, Tufts University School of Medicine, Boston, MA, USA; ^8^Wang Vision Institute, Nashville, TN, USA

## Abstract

**Purpose:**

To evaluate the efficacy of self-retained cryopreserved amniotic membrane (CAM) in promoting corneal nerve regeneration and improving corneal sensitivity in dry eye disease (DED).

**Methods:**

In this prospective randomized clinical trial, subjects with DED were randomized to receive CAM (study group) or conventional maximum treatment (control). Changes in signs and symptoms, corneal sensitivity, topography, and in vivo confocal microscopy (IVCM) were evaluated at baseline, 1 month, and 3 months.

**Results:**

Twenty subjects (age 66.9 ± 8.9) were enrolled and 17 completed all follow-up visits. Signs and symptoms were significantly improved in the study group yet remained constant in the control. IVCM showed a significant increase in corneal nerve density in the study group (12,241 ± 5083 *μ*m/mm^2^ at baseline, 16,364 ± 3734 *μ*m/mm^2^ at 1 month, and 18,827 ± 5453 *μ*m/mm^2^ at 3 months, *p* = 0.015) but was unchanged in the control. This improvement was accompanied with a significant increase in corneal sensitivity (3.25 ± 0.6 cm at baseline, 5.2 ± 0.5 cm at 1 month, and 5.6 ± 0.4 cm at 3 months, *p* < 0.001) and corneal topography only in the study group.

**Conclusions:**

Self-retained CAM is a promising therapy for corneal nerve regeneration and accelerated recovery of the ocular surface health in patients with DED. The study is registered at clinicaltrials.gov with trial identifier: NCT02764814.

## 1. Introduction

Dry eye disease (DED) is a multifactorial disease of the tears and ocular surface that results in symptoms of discomfort, visual disturbance, and tear film instability [1]. Despite different underlying pathogenic processes, inflammation is a common denominator in DED, which in turn induces further damage to the corneal epithelium and its underlying nerve plexus [2]. These nerves are responsible for regulating the corneal sensitivity, blink reflex, tear production, and epithelial regeneration, and hence, its injury further induces a self-perpetuating cycle of deterioration [3]. In fact, recent studies have demonstrated the pathological role of corneal nerve dysfunction in many ocular surface disorders including DED. They demonstrated a strong correlation between the severity of DED and the loss of corneal nerves, suggesting that corneal nerve density can be used to gauge the severity of DED [4–6]. Furthermore, DED has been associated with a change of inflammatory cell count, thus confirming its inflammatory nature [7]. Progress has been made in the diagnosis of corneal nerve loss using *in vivo* confocal microscopy (IVCM) [8–11]; however, therapeutic strategies aimed at restoring the damaged nerves are limited.

Cryopreserved amniotic membrane (CAM) has recently been used to treat DED with ocular surface involvement, and its efficacy has been attributed to its known potent anti-inflammatory effect [12]. However, CAM is also rich in neurotrophic factors, particularly nerve growth factor (NGF), which may promote corneal nerve regeneration and hence explain its lasting effect in DED treatment [13–15]. Therefore, in this study, we used IVCM to investigate the potential effect of self-retained CAM on corneal nerve regeneration in patients with DED and correlate this effect to change in dry eye signs and symptoms, corneal topography, and corneal sensitivity.

## 2. Methods

### 2.1. Study Design and Participants

This is a prospective randomized controlled study to evaluate the efficacy of self-retained CAM in restoring corneal nerve density and improving corneal sensitivity in patients with dry eye disease (DED). The study was approved by the Western Institutional Review Board (WIRB, Puyallup, WA) and conducted at the Thomas John Vision Institute (Tinley Park, Illinois, USA) in accordance with the Health Insurance Portability and Accountability Act (HIPAA) and Declaration of Helsinki. The study is registered at clinicaltrials.gov with trial identifier: NCT02764814.

Prior to participating in the study, a written informed consent was obtained from all subjects and each subject was assigned a unique subject ID. A detailed medical and ocular history was then obtained including interim medical conditions and current and previous treatments of DED. All subjects also underwent complete ophthalmic examination at baseline to determine their eligibility for the study. Inclusion criteria included subjects 21 years and older who had moderate to severe DED, grades 2–4, as defined by the Report of the International Dry Eye Work Shop (DEWS) [1]. Exclusion criteria included eyelid abnormality, symblepharon, active ocular allergies, known intolerances to CAM (present in PROKERA Slim (PKS) (Bio-Tissue, Inc., Miami, FL)), previous brain surgery or trigeminal nerve damage, recent ocular infection within 14 days, recent ocular surgery or injury within 3 months, contact lens wearers, pregnancy, concomitant therapy that affect tear function or ocular surface integrity, or subjects engaged in another ongoing clinical trial.

After meeting the eligibility criteria, twenty consecutive subjects were randomly assigned to the study group receiving PKS in one eye (*n* = 10) or the control group using conventional treatment (*n* = 10). To avoid potential contralateral effect, only one eye per subject was evaluated. Randomization was performed using a 10-block design at randomize.com.

### 2.2. Treatment Procedure

For the study group, PKS was inserted in the office under topical anesthesia with 0.5% proparacaine hydrochloride eye drops. After placement, the subjects were asked to continue topical medications as needed and return 3–5 days later to remove the PKS. Subjects in the control group were asked to continue their conventional maximum treatment throughout the duration of the study including artificial tears, cyclosporine A, serum tears, antibiotics, steroids, and nonsteroidal anti-inflammatory medications. All subjects returned at 1 and 3 months for clinical evaluation.

### 2.3. Clinical Evaluation

All subjects underwent complete ophthalmic evaluations, which included the following tests performed in the order of pain score, SPEED questionnaire [16], visual acuity, corneal topography, slit lamp examination, fluorescein staining (oxford scale), tear film breakup time (TFBUT), corneal sensitivity, Schirmer's test, MMP9 test, central corneal *in vivo* confocal microscopy, and DEWS scoring as detailed below.

#### 2.3.1. Pain Score

The pain score was measured subjectively using the Visual Analog Scale (VAS) ranging from 0 (none) to 10 (the worst) as previously described [17, 18].

#### 2.3.2. SPEED Questionnaire

Each participant underwent the validated Standard Patient Evaluation of Eye Dryness (SPEED) questionnaire, which is a frequency- and severity-based questionnaire designed to track long-term symptom changes over a period of 3 months [16, 19]. The composite score of the SPEED questionnaire is obtained by summing the scores from the frequency and severity of four symptoms of dry eye including (1) dryness or grittiness or scratchiness, (2) soreness or irritation, (3) burning or watering, and (4) eye fatigue. The frequency of each symptom was scored from 0 to 3: never (0), sometimes (1), often (2), and constant (3). Severity of each symptom was scored from 0 to 4: no problems (0), tolerable (1), uncomfortable (2), bothersome (3), or intolerable (4). The composite score is referred to as the “SPEED score” in the range of 0 to 28, with a higher score (28) indicating severe dry eye symptoms.

#### 2.3.3. Corneal Topography

**Figure 1:**
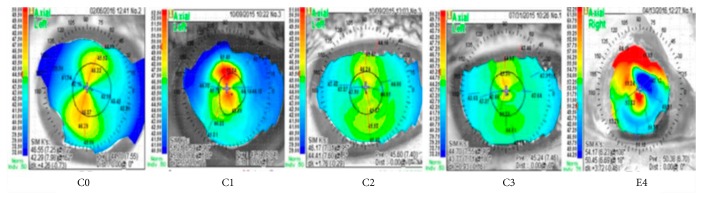
Representative images of Table 1.

Corneal topography was performed using Nidek OPD Scan ARK 10000 (Nidek Co. Ltd., Gamagori, Japan), and the test was conducted before applying any eye drops, and the subjects were asked to blink right before the images were obtained to avoid confounding effects. Images were analyzed by masked readers (NR, MW) at Wang Vision Institute, Nashville, TN, USA. The analyzed parameters included artificial steepness, wavefront errors, and total spherical aberrations. We further created a new grading scheme by classifying the shape of the topographic pattern as A (round), B (oval), C (symmetric bow tie), D (asymmetric bow tie), and E (irregular or unclassified), as previously described [20] and the regularity of the shape as 0 (no irregularity—best-predicted corrected vision), 1 (minimal irregularity), 2 (mild irregularity), 3 (moderate irregularity), or 4 (severe irregularity—worst-predicted corrected vision) (Table 1, Figure 1).

#### 2.3.4. Corneal Sensitivity

Corneal sensitivity was measured using the contact nylon thread Luneau 12/100 mm Cochet-Bonnet esthesiometer (Luneau; Prunay-Le-Gillon, France). The nylon filament was applied perpendicularly to the central cornea with no topical anesthetics. The filament length extends from 6 cm with 0.5 cm reduction intervals, and as the length decreases, the pressure increases. The corneal sensitivity was indicated as the longest filament length (cm) resulting in a positive response which was verified twice and recorded [21, 22].

#### 2.3.5. Detection of MMP-9

InflammaDry Detector (InflammaDry®; Rapid Pathogen Screening Inc., Sarasota, FL) was used to measure the level of MMP-9 prior to instilling ocular anesthetic, fluorescein, or Schirmer testing. Per manufacturer's recommendation, the sampling fleece was dabbed in multiple locations along the lower palpebral conjunctiva for saturation with tear fluid followed by immersion of the absorbent tip in the buffer vial for 30 sec. The results were then read after 10 minutes: 2 blue lines indicated a negative result (MMP-9 level < 40 ng/ml) whereas a blue line and red line indicated a positive result (MMP-9 ≥ 40 ng/ml).

#### 2.3.6.* In Vivo* Confocal Microscopy

Laser IVCM assessments were performed on the central cornea using HRT III/RCM (Heidelberg Engineering GmbH, Heidelberg, Germany). The examination was performed under topical anesthesia using an immersion lens (Olympus, Hamburg, Germany), with a magnification of ×60, and a contact objective covered by a disposable, sterile polymethyl methacrylate cap (Tomo-Cap; Heidelberg Engineering GmbH, Heidelberg, Germany) filled with a layer of hydroxypropyl methylcellulose 2.5% (GenTeal Gel; Novartis Ophthalmics, East Hanover, NJ). One drop of hydroxypropyl methylcellulose 2.5% was also placed in the patient eye and on the outside of the cap. The central cornea was scanned using sequence scan mode by a masked operator at a speed of 30 frames per second, and 40 coronal section images of 400 × 400 *μ*m were obtained.

The analysis of the images was performed by two masked readers (PH, ZMS) at the Boston Image Reading Center (Boston, MA) using the semiautomated tracing program NeuronJ, a plug-in for ImageJ software (developed by Wayne Rasband, National Institutes of Health, Bethesda, MD; available at http://imagej.nih.gov/ij/) as previously described [23]. The analysis was focused on the subbasal nerve plexus, that is, immediate images beneath the basal epithelium and/or anterior to the Bowman's layer. Three representative images per scan were selected by the masked readers for quantitative assessment of the density of subbasal nerves and dendritiform cells (DCs). Subbasal corneal nerve density was defined as the total length of the nerves visible within an image frame (expressed in *μ*m/mm^2^) and the DC density was expressed in cells/mm^2^.

#### 2.3.7. Grading of DEWS Score

The DEWS score was used to grade the severity of DED from 1 (none to mild) to 4 (severe) [1, 24]. The severity and frequency of DED symptoms including ocular discomfort, visual symptoms, conjunctival injection and staining, corneal staining, corneal/tear signs, lid/Meibomian glands, Schirmer's test, and TFBUT test were graded on a 4-point scale ranging from level 1 (mild and/or episodic) to level 4 (severe and/or disabling and constant).

### 2.4. Statistical Analysis

The sample size was calculated to detect an increase in corneal nerve density (primary outcome) of 5600 *μ*m/mm^2^ (SD = 3500 *μ*m/mm^2^) in the actively treated group over that in the control group [6]. Assuming comparison of posttreatment minus baseline differences between the groups with the two-tailed two-sample *t*-test, 7 patients per group would provide 80% power. The sample size was increased to 10 per group to compensate for possible dropouts. Descriptive statistics for continuous variables are reported as the mean ± SD and were analyzed using SPSS software, version 24.0 (SPSS Inc., Chicago, Illinois, USA). Differences between parameters before and after treatment were analyzed by the ANOVA test, student *t*-test, and the Wilcoxon signed-ranks test. Correlation between parameters was analyzed by the Spearman's rank order correlation. A *p* value less than 0.05 was considered statistically significant.

## 3. Results

In this study, 20 subjects (6 males, 14 females; age 66.9 ± 8.9) with moderate to severe DED were enrolled and equally randomized into the study or control group (*n* = 10/group). A total of 17 patients completed all follow-up visits (4 males, 13 females; age 67.8 ± 8.9) with 9 in the study group (2 males, 7 females; age 64.8 ± 10.3) and 8 in the control group (2 males, 6 females; age 67.8 ± 9.8). In the study group, PKS was placed for 3.4 ± 0.7 days (ranging 3–5 days).

### 3.1. Symptoms and Signs

**Figure 2:**
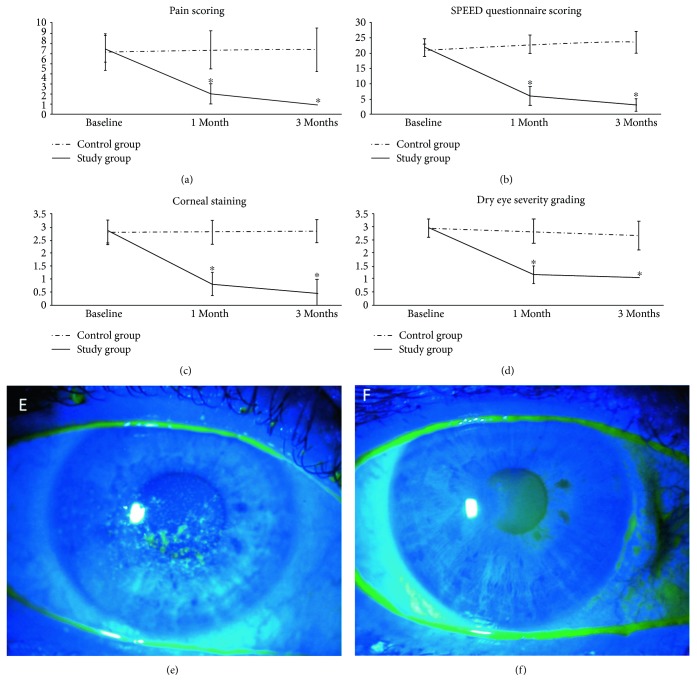
Changes in DED severity: pain score (a), SPEED score (b) corneal staining score (c), and DEWS score (d) and an illustrative example of fluorescein staining before (e) and after (f) PKS treatment. Significant decrease in pain score, SPEED questionnaire score, and symptoms in the study group (solid lines) from baseline to 3 months (*p* ≤ 0.001), while remained relatively unchanged in the control group (dash lines). ∗ denotes *p* ≤ 0.05.

The overall dry eye symptoms including discomfort and visual disturbances were significantly improved in the study group over the course of the study yet remained constant in the control group. In the study group, the pain score decreased significantly from 7.1 ± 1.5 at baseline to 2.2 ± 1.1 at 1 month and 1.0 ± 0.0 at 3 months (*p* ≤ 0.001, Figure 2(a)). Similarly, the SPEED questionnaire showed a marked decrease from 21.8 ± 3.2 at baseline to 5.9 ± 3.1 at 1 month and 2.8 ± 1.9 at 3 months (*p* ≤ 0.001) (Figure 2(b)). Consistent with the reduced symptoms, corneal staining had reduced significantly from 2.8 ± 0.4 at baseline to 0.8 ± 0.4 at 1 month and 0.6 ± 0.5 at 3 months (*p* ≤ 0.001) (Figures 2(c), 2(e), and 2(f)). Although the Schirmer I test did not show significant improvement, TFBUT improved significantly from 8.3 ± 2.5 sec at baseline to 13.9 ± 2.2 sec at 1 month and 15.0 sec at 3 months (*p* ≤ 0.001). In contrast, subjects in the control group showed unchanged pain score (6.8 ± 1.9, 7 ± 2.1, and 7.3 ± 2, *p* = 0.4), SPEED (21.8 ± 2.1, 22.8 ± 3.1, and 23.3 ± 3, *p* = 0.06), fluorescein staining (2.8 ± 0.5, 2.8 ± 0.5, and 2.9 ± 0.4, *p* = 0.7), and TFBUT (8.1 ± 3.7, 8.8 ± 3.5, and 7.1 ± 3.9, *p* = 0.2) at baseline, 1 month, and 3 months, respectively.

Although there was an improvement in visual symptoms and the quality of vision in the study group compared to the control group, there was no statistical differences in the visual acuity from the baseline in both the study (0.36 ± 0.4, 0.32 ± 0.4, and 0.3 ± 0.4 logMAR) and control groups (0.35 ± 0.4, 0.27 ± 0.4, and 0.2 ± 0.3 logMAR) at baseline, 1 month, and 3 months, respectively. Additionally, there was no difference in the InflammaDry test results between groups; 3 (30%) subjects were positive for MMP9 at baseline in each group and turned negative thereafter. The results were not consistent with the severity of the signs and symptoms, consistent with prior reports, which may be due to prior use of anti-inflammatory medications [25, 26].

### 3.2. DEWS Score

Consistent with the aforementioned findings, the DEWS scoring was significantly reduced from 2.9 ± 0.3 at baseline to 1.1 ± 0.3 at 1 month and 1.0 at 3 months (*p* ≤ 0.001). In the control group, there was no statistical difference in the DEWS scoring at baseline (2.9 ± 0.4), 1 month (2.8 ± 0.5), and 3 months (2.7 ± 0.5) (*p* = 0.7) (Figure 2(d)).

### 3.3. Corneal Topography

**Figure 3:**
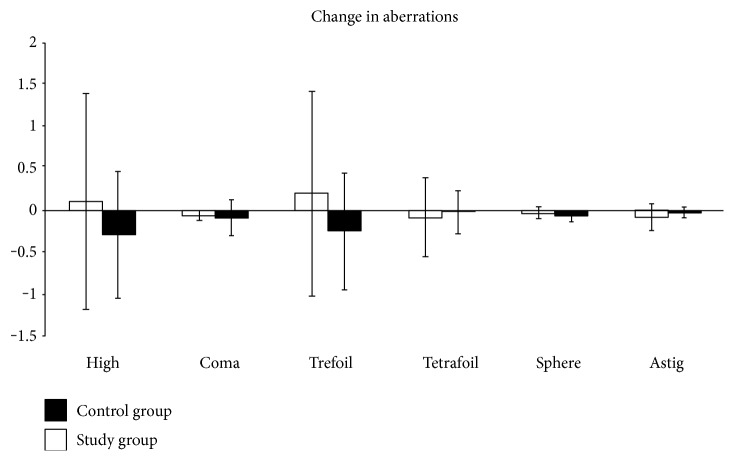
Changes in corneal topography from baseline to 3 months (*p* > 0.05). It showed decreased high-order aberration in the study group but not in the control group.

Corneal topography, read in the masked fashion, showed decreased high-order aberrations (HOA) in the study group from baseline to 3 months (−0.3 ± 0.8). HOA got worse in the control group from baseline to 3 months (0.1 ± 1.3) (Figure 3). However, there were no statistically significant differences in total aberrations in either the study (*p* = 0.4) or control (*p* = 0.6). Keratometric astigmatism (ΔK) was slightly reduced in the study group from 2.1 ± 1.8 at baseline to 1.9 ± 1.8 (*p* = 0.9) at 3 months, and wavefront error was also improved from 1.03 at baseline to 0.3 (*p* = 0.3) at 3 months. Both artificial steepening (ΔK) and wavefront error remained relatively the same in the control group [1.9 to 2 (*p* = 0.9) and 0.9 to 0.7 (*p* = 0.5) at baseline and 3 months, resp.].

Corneal regularity slightly improved in the study group from 1.9 ± 1.1 at baseline to 1.6 ± 0.9 at 1 month and 1.7 ± 0.9 at 3 months; however, the difference was not statistically significant (*p* = 0.7). In the control group, it was also improved from 2.5 ± 0.8 at baseline to 2.3 ± 0.9 and 1.6 ± 0.7 (*p* = 0.3) at 1 and 3 months, respectively. Likewise, the shape of the topographic pattern was not significantly different (*p* = 0.3) between the study and control groups (3.9 ± 0.8, 3.4 ± 0.9, and 3 ± 1.1 (*p* = 0.7) and 4.5 ± 0.5, 4.1 ± 0.6, and 3.9 ± 0.8 (*p* = 0.4) at baseline, 1, and 3 months, resp.).

### 3.4. Corneal Nerve and Dendritiform Cell Density

**Figure 4:**
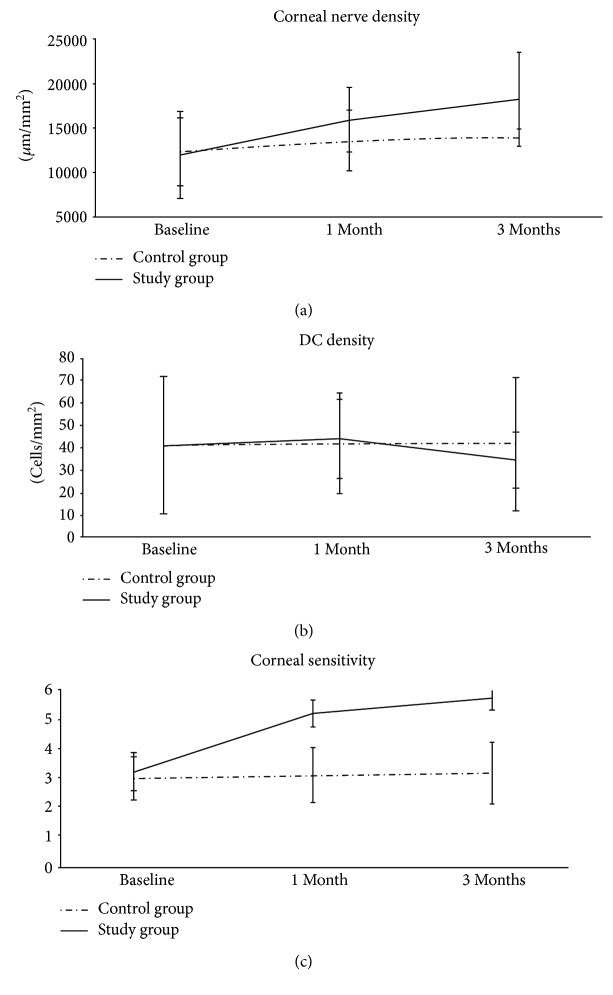
Changes in corneal nerve density (a), dendritiform cell density (b), and corneal sensitivity (c), in the study group (solid lines) and control group (dash lines). In the study group, there was a significant increase in the central corneal nerve density and a nonsignificant change in the dendritiform cell density. In the control group, both corneal nerve and dendritiform cell density remained relatively unchanged.

**Figure 5:**
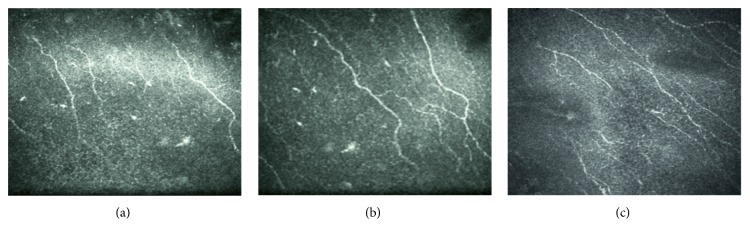
Illustrative example of IVCM showing the subbasal nerve fiber and dendritiform cells in the study group at baseline (a), 1 month (b), and 3 months follow-up (c).

In the study group, IVCM images read in the masked fashion showed a significant increase in the central corneal nerve density from 12,241 ± 5083 *μ*m/mm^2^ at baseline to 16,364 ± 3734 *μ*m/mm^2^ at 1 month and 18,827 ± 5453 *μ*m/mm^2^ at 3 months (*p* = 0.015) (Figures 4(a) and 5). This improvement was accompanied by a non-significant change in the DC density, that is, from 43.6 ± 32.2 cells/mm^2^ at baseline to 46.9 ± 18.5 at 1 month and then decreased to 36.9 ± 13.1 cells/mm^2^ at 3 months (*p* = 0.5) (Figure 4(b)). In the control group, the corneal nerve density remained relatively unchanged from 12,554 ± 3989 *μ*m/mm^2^ at baseline to 13,956 ± 3555 *μ*m/mm^2^ at 1 month and 14,254 ± 1055 *μ*m/mm^2^ at 3 months (*p* = 0.5). The DC density also remained relatively unchanged, that is, 43.8 ± 32.1 cells/mm^2^ at baseline, 44.7 ± 23.4 cells/mm^2^ at 1 month, and 44.2 ± 31.2 cells/mm^2^ at 3 months (*p* = 0.2).

### 3.5. Corneal Sensitivity

In the study group, there was a significant increase in corneal sensitivity from 3.25 ± 0.6 cm at baseline to 5.2 ± 0.5 cm at 1 month and 5.6 ± 0.4 cm at 3 months, (*p* < 0.001). This improvement was significantly correlated with the increase of corneal nerve density (*p* = 0.03, *r* = 0.44). In contrast, corneal sensitivity remained unchanged in the control group: 3.0 ± 0.7 cm at baseline, 3.2 ± 0.9 cm at 1 month, and 3.0 ± 0.9 cm at 3 months (*p* = 0.8, *r* = −0.08) (Figure 4(c)).

## 4. Discussion

This prospective randomized controlled clinical trial demonstrates that self-retained CAM through the placement of PKS can accelerate the recovery of corneal surface health that lasts for at least three months in patients with moderate and severe DED. This finding is consistent with our recently reported retrospective study [12]. Compared to the controls, single placement of PKS for 3.4 ± 0.7 days resulted in a significant improvement of DED symptoms and signs (Figures 2(a) and 2(b)), reduction of corneal surface irregularity (Figure 2(c)), and reduction of DEWS scoring (Figure 2(d)) at one month and three months. For the first time, we demonstrate that such a therapeutic efficacy is correlated with corneal nerve regeneration as evidenced by a significant increase in subbasal corneal nerve density (Figure 4(a)) and corneal sensitivity (Figure 4(c)). We thus surmise that the lasting effect of a single placement of PKS for treating DED may be attributed to corneal nerve regeneration. This interpretation is supported by the fact that corneal nerves play a vital role in epithelial regeneration and tear film stability through reflex tearing and blinking. It also explains why the aforementioned corneal nerve regeneration can restore corneal surface as evidenced by resolution of corneal punctuate staining and improvement of tear film stability (i.e., lengthened TFBUT), despite that the Schirmer I test did not show significant changes. Tear film instability and corneal epitheliopathy in DED are known causes of higher corneal aberrations [27, 28]. Indeed, corneal topography in our study also showed consistent improvement of the HOAs and trefoil aberrations in the study group as compared to those in the control group where there was progressive worsening although the difference was not significant (Figure 3()).

It has been well established that inflammation triggered by both innate and adaptive immune responses is critical to pathogenesis and the chronicity of DED [29, 30]. Recent IVCM studies have further shown an increase in DCs and a decrease of subbasal corneal nerve density in DED [7, 23, 31, 32]. Herein, we noted that a single placement of PKS for 3–5 days significantly increased the subbasal corneal nerve density, while reducing the DEWS score from 2.9 ± 0.3 at baseline to 1.1 ± 0.3 at 1 month and 1.0 at 3 months in the treatment group (*p* < 0.001), consistent with the notion that a decrease of subbasal nerve density is correlated with the severity of DED. We thus believe that CAM in PKS exerts a direct positive impact on the corneal nerve regeneration. This notion is supported by the finding that nerve growth factor (NGF) known to play an important role in nerve regeneration and epithelial healing [4, 15, 33] is abundantly present in CAM [13, 14]. However, autologous serum, which is reported to have a high concentration of NGF, [34] requires frequent and continuous use to produce an effect. Hence, additional factors may be involved. Recently, our laboratory has biochemically purified and characterized a unique matrix termed HC-HA/PTX3 from the amniotic membrane (AM) responsible for AM's anti-inflammatory, antiscarring, and antiangiogenic actions [35–41] (also reviewed in [42, 43]). Studies have shown the beneficial effect of amniotic membrane extract and HC-HA/PTX3 in DED animal models [44, 45]. Hence, future studies are needed to determine whether HC-HA/PTX3 also helps promote the increase of subbasal corneal nerve density.

Taking the anti-inflammatory action as an example, CAM has been demonstrated to induce apoptosis of neutrophils [46, 47], monocytes, and macrophages [48]; reduce infiltration of neutrophils, [46, 47], macrophages [49, 50], and lymphocytes, [51]; and promote polarization of M2 macrophages [52]. Such anti-inflammatory action exerted by CAM is retained in the water-soluble extract [53, 54] and replicated by HC-HA/PTX3 purified from AM [13, 14]. Collectively, the aforementioned anti-inflammatory actions might also help treating DED. On the other hand, conventional topical anti-inflammatory therapies such as cyclosporine [55], corticosteroids [56], or nonsteroidal anti-inflammatory drugs [57] not only can suppress inflammation but also compromise corneal nerves and subsequently delay corneal wound healing. For example, cyclosporine eye drops reduce cytokine expression in the cornea and retards regenerative sprouting from transected corneal stromal nerve trunks in an animal model [55]. Additionally, corticosteroids decreased the levels of tear NGF in patients with DED [56]. Despite our finding that CAM may promote the subbasal corneal nerve density, DC density was within the normal range in both groups. This may have been due to the fact that patients were on prior anti-inflammatory therapies, and thus, a minimal change was observed throughout the study. Other limitations of this study include the small sample size, potential placebo effect, and lack of IVCM at earlier time points. Further studies with a larger sample size and sham control group can further confirm these findings and determine the relationship between DC and the subbasal corneal nerve density in DED.

## 5. Conclusions

Self-retained CAM is a promising therapy for corneal nerve regeneration and accelerated recovery of the ocular surface health in patients with DED.

## Figures and Tables

**Table 1:** Grading of topographic pattern.

Shape	Regularity of shape
A: round	0: no irregularity
B: oval	1: minimal irregularity
C: symmetric bowtie	2: mild irregularity
D: asymmetric bowtie	3: moderate irregularity
E: unclassified	4: severe irregularity

## References

[1] (2007). The definition and classification of dry eye disease: report of the definition and classification subcommittee of the International Dry Eye WorkShop. *The Ocular Surface*.

[2] Sheha H., Tseng S. C. G. (2013). The role of amniotic membrane for managing dry eye disease. *Ocular Surface Disorder*.

[3] Shaheen B. S., Bakir M., Jain S. (2014). Corneal nerves in health and disease. *Survey of Ophthalmology*.

[4] Lambiase A., Sacchetti M., Bonini S. (2012). Nerve growth factor therapy for corneal disease. *Current Opinion in Ophthalmology*.

[5] Jain P., Li R., Lama T., Saragovi H. U., Cumberlidge G., Meerovitch K. (2011). An NGF mimetic, MIM-D3, stimulates conjunctival cell glycoconjugate secretion and demonstrates therapeutic efficacy in a rat model of dry eye. *Experimental Eye Research*.

[6] Labbé A., Liang Q., Wang Z. (2013). Corneal nerve structure and function in patients with non-sjogren dry eye: clinical correlations. *Investigative Ophthalmology & Visual Science*.

[7] Lin H., Li W., Dong N. (2010). Changes in corneal epithelial layer inflammatory cells in aqueous tear-deficient dry eye. *Investigative Ophthalmology & Visual Science*.

[8] Tuisku I. S., Konttinen Y. T., Konttinen L. M., Tervo T. M. (2008). Alterations in corneal sensitivity and nerve morphology in patients with primary Sjogren’s syndrome. *Experimental Eye Research*.

[9] Alhatem A., Cavalcanti B., Hamrah P. (2012). *In vivo* confocal microscopy in dry eye disease and related conditions. *Seminars in Ophthalmology*.

[10] Patel D. V., McGhee C. N. (2013). Quantitative analysis of in vivo confocal microscopy images: a review. *Survey of Ophthalmology*.

[11] Cruzat A., Qazi Y., Hamrah P. (2017). In vivo confocal microscopy of corneal nerves in health and disease. *The Ocular Surface*.

[12] Cheng A. M., Zhao D., Chen R. (2016). Accelerated restoration of ocular surface health in dry eye disease by self-retained cryopreserved amniotic membrane. *The Ocular Surface*.

[13] Touhami A., Grueterich M., Tseng S. C. (2002). The role of NGF signaling in human limbal epithelium expanded by amniotic membrane culture. *Investigative Ophthalmology & Visual Science*.

[14] Banerjee A., Nürnberger S., Hennerbichler S. (2014). In toto differentiation of human amniotic membrane towards the Schwann cell lineage. *Cell and Tissue Banking*.

[15] Aloe L., Tirassa P., Lambiase A. (2008). The topical application of nerve growth factor as a pharmacological tool for human corneal and skin ulcers. *Pharmacological Research*.

[16] Asiedu K., Kyei S., Mensah S. N., Ocansey S., Abu L. S., Kyere E. A. (2016). Ocular surface disease index (OSDI) versus the standard patient evaluation of eye dryness (SPEED): a study of a nonclinical sample. *Cornea*.

[17] Price D. D., Bush F. M., Long S., Harkins S. W. (1994). A comparison of pain measurement characteristics of mechanical visual analogue and simple numerical rating scales. *Pain*.

[18] Cui J. Z., Geng Z. S., Zhang Y. H., Feng J. Y., Zhu P., Zhang X. B. (2016). Effects of intracutaneous injections of sterile water in patients with acute low back pain: a randomized, controlled, clinical trial. *Brazilian Journal of Medical and Biological Research*.

[19] Ngo W., Situ P., Keir N., Korb D., Blackie C., Simpson T. (2013). Psychometric properties and validation of the standard patient evaluation of eye dryness questionnaire. *Cornea*.

[20] Bogan S. J., Waring G. O., Ibrahim O., Drews C., Curtis L. (1990). Classification of normal corneal topography based on computer-assisted videokeratography. *Archives of Ophthalmology*.

[21] Xu K.-P., Yagi Y., Tsubota K. (1996). Decrease in corneal sensitivity and change in tear function in dry eye. *Cornea*.

[22] Labbe A., Alalwani H., Van Went  C., Brasnu E., Georgescu D., Baudouin C. (2012). The relationship between subbasal nerve morphology and corneal sensation in ocular surface disease. *Investigative Ophthalmology & Visual Science*.

[23] Cruzat A., Witkin D., Baniasadi N. (2011). Inflammation and the nervous system: the connection in the cornea in patients with infectious keratitis. *Investigative Ophthalmology & Visual Science*.

[24] Maychuk D. Y., Dry Eye Prevalence Study Group (2016). Prevalence and severity of dry eye in candidates for laser in situ keratomileusis for myopia in Russia. *Journal of Cataract and Refractive Surgery*.

[25] Lanza N. L., McClellan A. L., Batawi H. (2016). Dry eye profiles in patients with a positive elevated surface matrix metalloproteinase 9 point-of-care test versus negative patients. *The Ocular Surface*.

[26] Messmer E. M., von Lindenfels V., Garbe A., Kampik A. (2016). Matrix metalloproteinase 9 testing in dry eye disease using a commercially available point-of-care immunoassay. *Ophthalmology*.

[27] Montes-Mico R., Caliz A., Alio J. L. (2004). Wavefront analysis of higher order aberrations in dry eye patients. *Journal of Refractive Surgery*.

[28] Koh S., Maeda N., Hirohara Y. (2008). Serial measurements of higher-order aberrations after blinking in patients with dry eye. *Investigative Ophthalmology & Visual Science*.

[29] Wei Y., Asbell P. A. (2014). The core mechanism of dry eye disease is inflammation. *Eye & Contact Lens*.

[30] Yagci A., Gurdal C. (2014). The role and treatment of inflammation in dry eye disease. *International Ophthalmology*.

[31] Kheirkhah A., Rahimi Darabad R., Cruzat A. (2015). Corneal epithelial immune dendritic cell alterations in subtypes of dry eye disease: a pilot in vivo confocal microscopic study. *Investigative Ophthalmology & Visual Science*.

[32] Benítez-Del-Castillo J. M., Acosta M. C., Wassfi M. A. (2007). Relation between corneal innervation with confocal microscopy and corneal sensitivity with noncontact esthesiometry in patients with dry eye. *Investigative Ophthalmology & Visual Science*.

[33] Lambiase A., Mantelli F., Sacchetti M., Rossi S., Aloe L., Bonini S. (2011). Clinical applications of NGF in ocular diseases. *Archives Italiennes de Biologie*.

[34] Matsumoto Y., Dogru M., Goto E. (2004). Autologous serum application in the treatment of neurotrophic keratopathy. *Ophthalmology*.

[35] He H., Li W., Tseng D. Y. (2009). Biochemical characterization and function of complexes formed by hyaluronan and the heavy chains of inter-alpha-inhibitor (HC^∗^HA) purified from extracts of human amniotic membrane. *The Journal of Biological Chemistry*.

[36] Zhang S., He H., Day A. J., Tseng S. C. (2012). Constitutive expression of inter-alpha-inhibitor (IalphaI) family proteins and tumor necrosis factor-stimulated gene-6 (TSG-6) by human amniotic membrane epithelial and stromal cells supporting formation of the heavy chain-hyaluronan (HC-HA) complex. *The Journal of Biological Chemistry*.

[37] Blom A. M., Morgelin M., Oyen M., Jarvet J., Fries E. (1999). Structural characterization of inter-alpha-inhibitor. Evidence for an extended shape. *The Journal of Biological Chemistry*.

[38] Zhuo L., Hascall V. C., Kimata K. (2004). Inter-alpha-trypsin inhibitor, a covalent protein-glycosaminoglycan-protein complex. *The Journal of Biological Chemistry*.

[39] Shay E., He H., Sakurai S., Tseng S. C. (2011). Inhibition of angiogenesis by HC.HA, a complex of hyaluronan and the heavy chain of inter-alpha-inhibitor, purified from human amniotic membrane. *Investigative Ophthalmology & Visual Science*.

[40] He H., Zhang S., Tighe S., Son J., Tseng S. C. (2013). Immobilized heavy chain-hyaluronic acid polarizes lipopolysaccharide-activated macrophages toward M2 phenotype. *The Journal of Biological Chemistry*.

[41] He H., Tan Y., Duffort S., Perez V. L., Tseng S. C. (2014). In vivo downregulation of innate and adaptive immune responses in corneal allograft rejection by HC-HA/PTX3 complex purified from amniotic membrane. *Investigative Ophthalmology & Visual Science*.

[42] Tseng S. C. G. (2015). HC-HA/PTX3 purified from amniotic membrane as novel regenerative matrix: insight into relationship between inflammation and regeneration. *Investigative Ophthalmology & Visual Science*.

[43] Tseng S. C., He H., Zhang S., Chen S. Y. (2016). Niche regulation of limbal epithelial stem cells relationship between inflammation and regeneration. *The Ocular Surface*.

[44] Xiao X., Luo P., Zhao H. (2013). Amniotic membrane extract ameliorates benzalkonium chloride-induced dry eye in a murine model. *Experimental Eye Research*.

[45] Ogawa Y., He H., Mukai S. (2017). Heavy chain-hyaluronan/pentraxin 3 from amniotic membrane suppresses inflammation and scarring in murine lacrimal gland and conjunctiva of chronic graft-versus-host disease. *Scientific Reports*.

[46] Park W. C., Tseng S. C. G. (2000). Modulation of acute inflammation and keratocyte death by suturing, blood and amniotic membrane in PRK. *Investigative Ophthalmology & Visual Science*.

[47] Wang M. X., Gray T. B., Park W. C. (2001). Corneal haze and apoptosis is reduced by amniotic membrane matrix in excimer laser photoablation in rabbits. *Journal of Cataract & Refractive Surgery*.

[48] Shimmura S., Shimazaki J., Ohashi Y., Tsubota K. (2001). Antiinflammatory effects of amniotic membrane transplantation in ocular surface disorders. *Cornea*.

[49] Bauer D., Wasmuth S., Hermans P. (2007). On the influence of neutrophils in corneas with necrotizing HSV-1 keratitis following amniotic membrane transplantation. *Experimental Eye Research*.

[50] Heiligenhaus A., Bauer D., Meller D., Steuhl K. P., Tseng S. C. (2001). Improvement of HSV-1 necrotizing keratitis with amniotic membrane transplantation. *Investigative Ophthalmology & Visual Science*.

[51] Bauer D., Wasmuth S., Hennig M., Baehler H., Steuhl K. P., Heiligenhaus A. (2009). Amniotic membrane transplantation induces apoptosis in T lymphocytes in murine corneas with experimental herpetic stromal keratitis. *Investigative Ophthalmology & Visual Science*.

[52] Bauer D., Hennig M., Wasmuth S. (2012). Amniotic membrane induces peroxisome proliferator-activated receptor-gamma positive alternatively activated macrophages. *Investigative Ophthalmology & Visual Science*.

[53] Li W., He H., Kawakita T., Espana E. M., Tseng S. C. G. (2006). Amniotic membrane induces apoptosis of interferon-gamma activated macrophages in vitro. *Experimental Eye Research*.

[54] He H., Li W., Chen S. Y. (2008). Suppression of activation and induction of apoptosis in RAW264.7 cells by amniotic membrane extract. *Investigative Ophthalmology & Visual Science*.

[55] Namavari A., Chaudhary S., Chang J. H. (2012). Cyclosporine immunomodulation retards regeneration of surgically transected corneal nerves. *Investigative Ophthalmology & Visual Science*.

[56] Lee H. K., Ryu I. H., Seo K. Y., Hong S., Kim H. C., Kim E. K. (2006). Topical 0.1% prednisolone lowers nerve growth factor expression in keratoconjunctivitis sicca patients. *Ophthalmology*.

[57] Gaynes B. I., Onyekwuluje A. (2008). Topical ophthalmic NSAIDs: a discussion with focus on nepafenac ophthalmic suspension. *Clinical Ophthalmology*.

